# Primary Adrenal Lymphoma with Secondary Central Nervous System Involvement: A Case Report and Review of the Literature

**DOI:** 10.4274/Tjh.2012.0172

**Published:** 2013-12-05

**Authors:** Kübra Aydın, Kerem Okutur, Mustafa Bozkurt, Özlem Aydın, Esat Namal, Akın Öztürk, Kezban Nur Pilancı, Reyhan Diz Küçükkaya, Osman Gökhan Demir

**Affiliations:** 1 İstanbul Bilim University Division of Medical Oncology, Department of Internal Medicine, İstanbul, Turkey; 2 İstanbul Bilim University Division of Hematology, Department of Internal Medicine, İstanbul, Turkey; 3 Acıbadem University School of Medicine, Department of Pathology, İstanbul, Turkey

**Keywords:** Primary adrenal lymphoma, Central nervous system involvement

## Abstract

Approximately 10%-20% of all systemic lymphomas have central nervous system (CNS) involvement, which has been correlated to a worsened prognosis. It is well known that secondary involvement of the adrenal glands may occur in up to 25% of patients during the course of diffuse lymphoma. Primary adrenal lymphoma (PAL), however, is a different entity, and it is defined as the presence of adrenal lymphoma without evidence of either nodal involvement or leukemia. It has been shown that this occurrence is rarely accompanied by extranodal involvement, such as in the CNS. PAL exhibits a tendency for CNS relapse and this possibility should be examined even before symptoms are present. Herein we present a patient with PAL and secondary CNS involvement.

## Abstract (tr)

Tüm sistemik lenfomaların yaklaşık %10-20’sinde merkezi sinir sistemi (MSS) tutulumu mevcuttur ve bunun kötü prognozla ilişkili olduğu görülmüştür. Yaygın lenfoma hastalık seyrinde hastaların %25’inde ikincil adrenal bez tutulumu olduğu iyi bilinmektedir. Fakat primer adrenal lenfoma (PAL) farklı bir kavramdır ve ne lösemi ne de lenf bezi tutulumu olmadan adrenal lenfoma görülmesi olarak tanımlanmıştır. Bu duruma nadiren MSS gibi lenf bezi dışı tutulumların eşlik ettiği görülmüştür. Sonuç olarak PAL MSS’de nüks etme eğilimi göstermektedir ve bu belirti vermeden önce araştılmalıdır. Burada ikincil MSS tutulumu olan bir PAL olgusu sunmaktayız.

## INTRODUCTION

Central nervous system (CNS) involvement is found in about 10%-20% of all systemic lymphomas, and it is generally correlated with a worsened prognosis [1]. Secondary involvement of the CNS in non-Hodgkin lymphoma may be shown in several different ways, and rapid control of CNS involvement in this scenario is deemed to be necessary to prevent neurologic morbidity and to preserve/enhance the quality of life. Lymphoma cells seem to enter the CNS via hematogenous spread or direct extension from adjacent bone metastases, or through centripetal growth along neurovascular bundles. It has also been hypothesized that known lymphoma cells have a potential to spread from retroperitoneal lymph nodes or patient’s bone marrow to the leptomeninges via the intervertebral venous plexus [2]. 

Several series of autopsies have shown that up to 25% of patients with non-Hodgkin lymphoma have adrenal gland involvement, and this is usually associated with advanced disease [3]. However, in contrast, primary adrenal lymphoma (PAL) is a different entity, defined as the presence of adrenal lymphoma without evidence of nodal involvement or leukemia. In addition, this has been found to be rarely accompanied by extranodal involvement. This is considered to be an enigma, however, because a normal adrenal gland in humans has been shown to be devoid of any lymphoid or hematopoietic tissue. Possible explanations for the occurrence of PAL include either preexisting autoimmune adrenalitis with lymphocyte infiltration or hematopoietic rest tissue in the adrenals, although this is not conclusively proven to exist due to the rarity of the disease [4]. 

In this article we report a rare case of PAL with secondary CNS involvement and review the literature. 

## CASE REPORT

**Figure 1:**
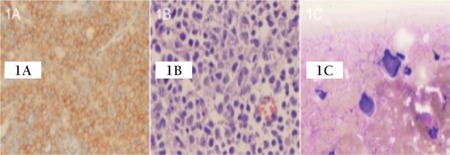
The histopathological examination of the specimen showed a.CD20(+) large B cell lymphoma

**Figure 2:**
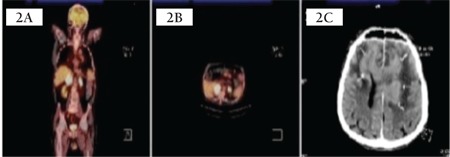
The images of PET-CT and Cranial MR

A 75-year-old male was admitted to our neurology clinic with a chief complaint of dullness for a duration of 2 months. The patient had a history of diabetes mellitus and Parkinson’s disease. A brain MRI was obtained and revealed a paramedian subcortical mass measuring 30 mm x 25 mm x 25 mm, located on the superior and middle frontal gyrus of the left brain hemisphere. Moreover, this lesion was accompanied by diffuse perilesional vasogenic edema. Due to the mass and edema effects, there was compression noted on the anterior horn of the left lateral ventricle. Additionally, a similar mass was detected measuring 2.5 cm in diameter located at the right frontal periventricular. After diagnosis, the patient underwent surgical intervention and an excisional biopsy was performed. The histopathological examination of the specimen revealed a CD20+ large B cell lymphoma (Figures 1A and 1B). Subsequently, the patient was referred to our medical oncology outpatient clinic. Physical examination at the outpatient admission showed no pathological findings correlated to vital signs and systems. The patient’s blood pressure was 120/70 mmHg, while the respiratory rate was 18/min, pulse rate was 67/min, and body temperature was 37.0 °C. There was no lymphadenopathy or organomegaly noted. Laboratory tests revealed a normal complete blood count; however, the erythrocyte sedimentation rate was found to be 45 mm/h. Additionally, β2-microglobulin and other laboratory values were found to be within normal limits. The patient’s baseline lactate dehydrogenase (LDH) level was within the normal levels. HIV test results were negative, while the International Prognostic Index score was calculated to be 4 (high risk). Chest computed tomography (CT) imaging was reviewed and determined to be normal, without any evidence of hilar lymphadenopathy or pulmonary lesions. An abdominal CT scan revealed bilateral adrenal mass. The left adrenal mass was measured as 30 mm x 33 mm, whereas the right adrenal mass was 32 mm x 53 mm in diameter. PET/CT scanning revealed a fluorodeoxyglucose (FDG) uptake focus only in the adrenal glands, and the brain was without pathologic glycolytic activity, as were all other regions of the body (Figures 2A and 2B). A CT-guided fine needle aspiration biopsy was obtained from both adrenal masses, and this scan revealed a CD20+ large B cell lymphoma similar in presentation to the patient’s brain pathology (Figure 1C). Bone marrow biopsy was normal, having no lymphoma infiltration. Thus, the diagnosis for this patient was reported to be stage IV diffuse large B cell PAL with secondary CNS involvement. Unfortunately, because of generalized seizures and status epilepticus, the patient was transferred to the intensive care unit (ICU). Antiepileptic and antiedema drugs were administered. Along the course, gram-negative septicemia occurred, and suitable antibiotics for coverage were included in the patient’s treatment. A new cranial CT was performed and suggested a rapid, progressive cranial mass (Figure 2C). Whole-brain radiotherapy was then initiated. Several days after initiation of radiotherapy, the patient improved, and he was subsequently transferred to a regular nursing floor. Whole-brain radiotherapy was completed, and then the patient was discharged from the hospital. Six weeks later, a repeat cranial MRI showed tumor regression. Afterwards, the patient was started on chemotherapy utilizing R-COP (rituximab 375 mg/m^2^, cyclophosphamide 750 mg/m^2^, vincristine 1.4 mg/m^2^, prednisone 100 mg/m^2^). We did not administer adriamycin due to the patient’s age, his general health status, his history of sepsis, and an increased risk of further infection. Unfortunately, 2-3 days after the completion of the third cycle of chemotherapy, the patient had a focal epileptic seizure. Repeat cranial MRI revealed a progressive left cranial mass, which at this time crossed the corpus callosum and was in the left side of the brain hemisphere. The patient was then readmitted to the ICU. Because of his age and history of whole-brain radiotherapy, a high dose of methotrexate could not be given. The patient died at the end of a 6 months of follow-up period due to disease progression. Informed consent was obtained. 

## DISCUSSION

Primary CNS lymphomas (PCNSLs) make up approximately 1% of intracranial neoplasms and only about 1% of extranodal non-Hodgkin lymphomas. These lymphomas are mostly present in individuals over 60 years of age, and this seems to be related to a reduction of immunologic vigilance, particularly T lymphocytes. PCNSL has a tendency to remain within the CNS and there is low incidence of systemic spread from these neoplasms [5]. In the case presented, we initially expected the outcome to be PCNSL; however, FDG uptake was detected in the bilateral adrenal glands in PET/CT, and the subsequent biopsy revealed a different diagnosis.

Bilateral adrenal masses are found to exist in about 10%-15% of the adrenal “incidentaloma” cases, likely to be diagnosed as metastatic disease (mostly from lung or breast), congenital adrenal hyperplasia, lymphoma, infection, hemorrhage, adrenocorticotropic hormone-dependent Cushing’s syndrome, and lastly, pheochromocytoma [6]. There are 2 types of adrenal involvement in lymphoma: PAL, which is defined as a disease originating from and confined to the adrenal glands solely, and non-Hodgkin lymphoma with adrenal involvement [7]. PAL is extremely rare; only about 100 cases have been previously reported in the literature [8]. PAL usually does not have disease found elsewhere, but if it is present, it is more likely extranodal in nature. Sites for extranodal involvement for PAL are the CNS and the gastrointestinal tract, as well as other endocrine organs [9]. In our case, CNS involvement was present. Mantzios et al. reviewed a total of 100 cases that had been previously reported in the literature over the past 4 decades [8]. According to their findings, PAL shows a predilection for older males with a male-to-female ratio of 2:1. The mean age at presentation with PAL was 65 years of age. These findings were similar in our case. As in our case, more than two-thirds of patients had a significant bilateral enlargement of the adrenal glands. The prognosis of PAL is poor. More than 90% of patients died within 1 year of diagnosis. Overall survival time in our case from time of diagnosis was roughly 6 months. Recently, Kim et al. published a study investigating prognostic factors in primary diffuse large B cell lymphoma (DLBCL) of the adrenal gland. Contrary to prior reports, their data suggested that outcomes of PAL are encouraging when a regimen of R-CHOP is utilized and that achieving complete response after R-CHOP therapy is predictive of survival [10].

Similarly, 5 cases of PAL with CNS involvement have been reported in the literature [10,11,12,13,14]. Table 1 summarizes the features of these reported cases. All of the patients reported were males older than the age of 50 (median age: 65), and bilateral involvement was noted in all. In one case, a high-grade atypical Burkitt/Burkitt-like lymphoma histology was found, while all others were cases of DLBCL. Only one patient presented initially with brain symptoms comparable to those of our case. Patients in the study had recurrence in the brain after systemic chemotherapy regimen. Survival was noted to be a median time of 6 months from initial diagnosis.

Some patients are at an increased risk for developing CNS relapse, especially if involvement of specific extranodal sites (bone marrow, epidural, testes, paranasal sinuses, kidneys, adrenal glands, liver, and breast) is noted, or if there are 2 or more extranodal sites with elevated LDH. Lumbar puncture should be performed for initial evaluation in all suspected patients. Although optimal management of these patients is still under investigation, intrathecal methotrexate (MTX) has historically been the most common used regimen. It has been suggested that systemic intravenous MTX at a dose of 3500 mg/m2 followed by leucovorin rescue should be adopted for CNS prophylaxis in high-risk patients and that intrathecal therapy be considered only for those patients who are not able to tolerate systemic therapy [15,16].

For patients with presentation of parenchymal CNS involvement, systemic methotrexate (3-3, 5 g/m2) should be incorporated into the treatment regimen. Systemic MTX with leucovorin rescue has been safely incorporated into R-CHOP-21, with MTX administrated on day 15 of the 21-day cycle. Systemic MTX is the optimal treatment for isolated CNS relapse that involves the brain parenchyma, and long-term survival is possible in some patients. These patients do not appear to benefit from intrathecal treatment, and only symptomatic benefit is noted from radiation treatment [17]. More case studies and data on the treatments performed are necessary to develop a better picture of diagnostic procedures and treatment regimens that have maximum efficacy. 

## CONFLICT OF INTEREST STATEMENT

The authors of this paper have no conflicts of interest, including specific financial interests, relationships, and/ or affiliations relevant to the subject matter or materials included.

## Figures and Tables

**Table 1:**
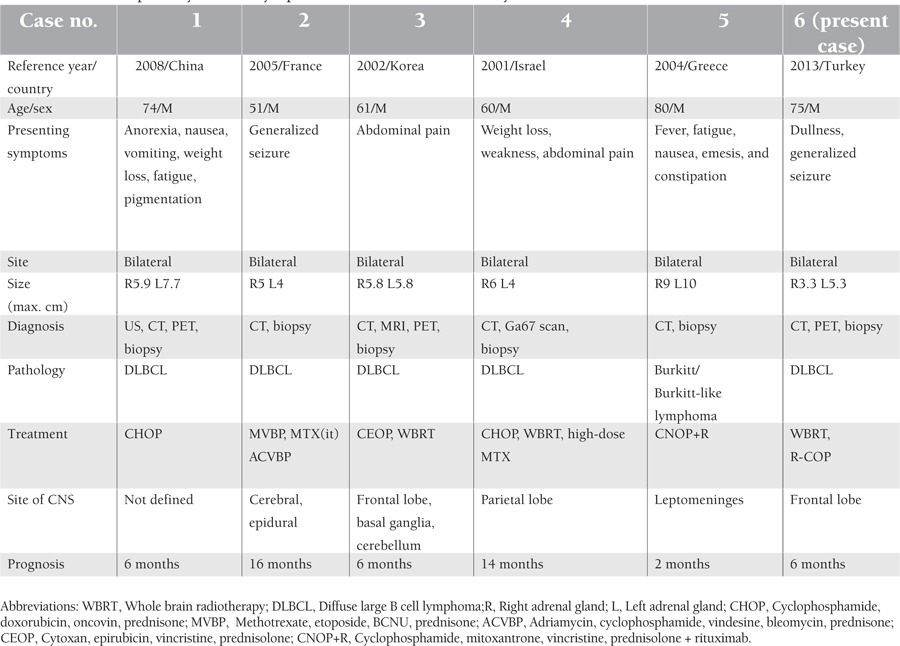
Cases of primary adrenal lymphoma with central nervous system involvement
